# The impact of strategic ventilation adjustments on stress responses in horses housed full-time in a vector-protected barn during the African horse sickness outbreak in Thailand

**DOI:** 10.1017/awf.2023.10

**Published:** 2023-03-23

**Authors:** Chanoknun Poochipakorn, Weena Joongpan, Pongphon Tongsangiam, Areeya Phooseerit, Kansuda Leelahapongsathon, Metha Chanda

**Affiliations:** 1 Veterinary Clinical Studies Program, Faculty of Veterinary Medicine, Kasetsart University Kamphaeng Saen Campus, Nakhon Pathom 73140, Thailand; 2 Faculty of Veterinary Medicine, Rajamangala University of Technology Tawan-Ok, Chonburi 20110, Thailand; 3 Department of Veterinary Public Health, Faculty of Veterinary Medicine, Kasetsart University, Kamphaeng Saen, Nakhon Pathom, 73140, Thailand; 4 Department of Large Animal and Wildlife Clinical Science, Faculty of Veterinary Medicine, Kasetsart University Kamphaeng Saen Campus, Nakhon Pathom 73140, Thailand; 5 Center of Veterinary Research and Academic Service, Faculty of Veterinary Medicine, Kasetsart University Bang Khen Campus, Bangkok 10900, Thailand

**Keywords:** African horse sickness, animal welfare, horse, strategic ventilation adjustment, stress, vector-protected barn

## Abstract

The severe outbreak of African horse sickness (AHS) in Thailand has forced horses to reside full-time inside barns that are covered by a small mesh net to prevent minuscule AHS insect vectors from gaining access. However, housing in the net-covered barn induces stress in horses, which compromises their welfare. Implementing strategic airflow adjustment while retaining the vector-protection characteristics has been proposed to help alleviate this problem. The present study aimed to investigate the effect of strategic ventilation adjustment on blood cortisol levels, heart rate and behaviour in horses in a vector-protected barn. Nine horses underwent two sequential stabling conditions: vector-protected barn housing and housing in a barn in which the air ventilation was explicitly adjusted. Heart rate was higher in the afternoon in horses housed in the barn without ventilation adjustment, whereas no change was observed in the barn with ventilation adjustment. The vector-protected housing increased the horses’ behavioural scores. Blood cortisol level declined over time, and an earlier decrease was detected at 1400h in the barn with ventilation adjustment. Although airflow adjustment did not appear to statistically alter the stress response in horses during housing in the vector-protected barn, an earlier decline in cortisol level alongside an unchanged heart rate in horses during the day may indicate the positive impact of ventilation adjustment within the vector-protected barn. With limited options to reduce stress or discomfort in horses, this strategic protocol could, at least in part, be applied to managing horses’ welfare during the AHS outbreak.

## Introduction

A growing area of interest in animal welfare is measuring how animals respond to living in specific controlled environments (Broom 1991). The well-being of animals is classified as freedom from five conditions: 1) Hunger and thirst; 2) Discomfort; 3) Pain, injury or illness; 4) Abnormal expression of behaviour; and 5) Fear and distress (Mormede *et al.* 2018). The welfare issue is paramount in horses, as they are increasingly utilised for specific purposes, including agriculture, hauling, recreation and sports (Minero & Canali 2009). Even though horses are social animals and live naturally with conspecifics (Carenzi & Verga 2009), they are stabled primarily in individual housing, rather than group systems (Minero & Canali 2009; Rose-Meierhöfer *et al.* 2010). Housing in separate boxes has been implemented to protect against natural threats and self-injury and allow the horse to have adequate rest (Minero & Canali 2009). Nevertheless, the use of individual housing can negatively impact the horses’ welfare in multiple ways such as stress-related behaviours, aggression against humans, and being unresponsive to the environment (Ruet *et al.* 2019).

In addition to the individual housing, improper housing management may also cause psychological and physiological stress in horses, attributable to the potentially harmful material accumulation within the stable (Mills & Clarke 2007). Specifically, housing air quality can affect short- and long-term animal welfare (Clarke *et al.* 1987; Holcombe *et al.* 2001). Ammonia, released by the interaction between microorganisms and urine or faeces, is the most common noxious gas found in horses’ stables (Curtis *et al.* 1996). A poorly ventilated horse barn with low ammonia clearance increases ammonia concentrations in the stable. This increase affects the respiratory system, causing conditions such as mucociliary damage and increasing mucus production (Mills & Clarke 2007). Furthermore, poor stable ventilation increases the horse’s risk of exposure to various pathogens, including dust, airborne microorganisms and other noxious gases, leading to chronic airway disease (Elfman *et al.* 2011). Hence, adequate ventilation is crucial in preserving the horse’s welfare during stable housing.

African horse sickness (AHS), a non-contagious, vector-borne disease, emerged in Thailand in March 2020 (King *et al.* 2020; Lu *et al.* 2020). Caused by infection with a virus of the genus *Orbivirus*, AHS is transmitted primarily by haematophagous midges of the genus *Culicoides* (Meiswinkel *et al.* 2000; Capela *et al.* 2003; Mellor & Hamblin 2004; Zientara *et al.* 2015). Restricted individual housing in a barn fully covered with a vector-protection net is highly recommended during a severe AHS outbreak. A net-covered barn accommodation has been reported to reduce the contact between horses and midges (Lincoln *et al.* 2015). Since the haematophagous biting midges are small (1–3 mm in length) (Mellor *et al.* 2000; Bartsch *et al.* 2009) and are a nuisance to equids (Purse *et al.* 2015), installation of extremely small mesh netting, coated with an approved insecticide, is preferable for vector protection (Baker *et al.* 2015). Netting has enabled a rapid reduction in AHS viral transmission and fatality rate (Baker *et al.* 2015). However, there are disadvantages to housing in a net-covered barn, including a dramatic decrease in air ventilation compared to an open barn (Webster *et al.* 1987). Decreased air ventilation that accompanies netting use, combined with living in a restricted area, could induce stress, consequently impairing horses’ welfare, as indicated by a recent study (Joongpan *et al.* 2021).

It is currently unclear how to reduce stress in the barn while retaining vector protection characteristics. Therefore, the present study aimed to investigate the effects of strategic stable ventilation adjustment on the stress parameters of horses housed full-time in a vector-protected barn. It was hypothesised that the strategic adjustment of stable ventilation would reduce stress in the horses and retain the barn’s vector-protection characteristics.

## Materials and methods

### Study animals

Nine healthy horses (four geldings and five mares, with a mean [± SEM] weight of 427.4 [± 14.2] kg and aged 16.2 [± 1.0] years, from the Horse Lover’s Club of Patum Thani Province, Thailand) were included in this study. The province is located in central Thailand, where the mean annual temperature is approximately 28–30°C. Once the AHS outbreak was declared, the horses were housed long term in separate 4 × 4 m stables within the 40 × 12 m (length × width) barn fully covered with 4.96 cm^–2^ vector-protection net (Pongchiangthong Plastic Netting, Samutsakorn, Thailand). In addition, a temporary vector-protected area connected to the barn’s front gate was established to facilitate elimination of polluted air and maintain a closed vector protection system (Figure S1). Commercial feed was provided three times daily with free access to clean water. Due to the large number of horses and the limited exercise area, each horse in the club was allowed to walk in the vector-protected arena for 20–30 min every other day.

### Ethics

All animal procedures were approved by the The Ethics Committee for Animal Experiments of Kasetsart University (ACKU63-VET-036).

### Experimental design

**Figure 1.**
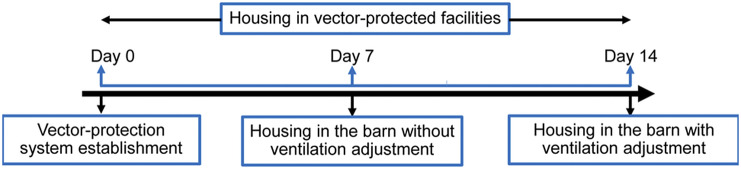
Diagram of the experimental procedure. The experiments were conducted at seven and 14 days after full-time housing in the vector-protected facilities.

**Figure 2.**
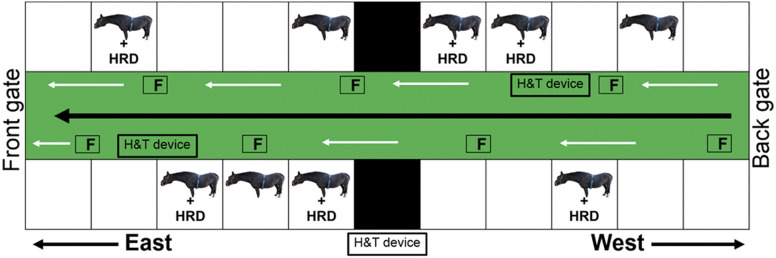
Individual horse housing in the vector-protected barn with ventilation adjustment. Six out of nine horses were equipped with a digital heart rate detector (HRD) for average heart-rate measurement. The floor-standing industrial fans (F) were placed in a zig-zag formation along the barn corridor. Humidity and temperature measuring devices (H&T devices) were installed inside and outside the barn.

The horses were housed full-time in the vector-protected facilities for six days prior to the commencement of the study. They experienced two housing conditions: housing in the net-covered barn without ventilation adjustment on the 7th day and housing in the net-covered barn with a strategic ventilation adjustment on the 14th day (Figure 1). Permanent fluorescent lighting with an average illuminance of 150 to 200 lux was provided for 12 h in the barn during the day and reduced to 50–100 lux at night. During the dates of the experiment, the horses did not undergo any exercise and were housed full-time in individual stables inside the vector-protected barn. Ventilation adjustment was performed by placing seven industrial floor-standing fans (63.5 cm in height, 800–1,200 revolutions per min) with the fan blades facing the barn corridor to produce one-way airflow to the front entrance opening (Figure 2). The goal was to maximise internal airflow during daytime. The air velocity was randomly determined within individual stables and across the barn corridor in both housing types using a Zephyrus Basic Anemometer (version 3.1.0, Gaia Consulting, Verona, Italy). The values were expressed as mean wind speed (m s^–1^) in the barn. Blood samples and relative data, including stable humidity and temperature, horse behaviour and heart rate (HR), were collected for 12 h (0600 to 1800h) in each housing type on alternate days. These data were compared within the period in each housing condition and between the two housing conditions.

### Stable humidity and temperature

Stable humidity and temperature were measured inside and outside the barn using a humidity data logger (TM-305U, Tenmars Electronics, Taipei, Taiwan). The installation of the humidity and temperature measuring devices is illustrated in Figure 2. Briefly, two devices were placed close to the central pathway inside the barn, and a third positioned outside the barn. Humidity and temperature were measured consecutively at 15-min intervals for the 12-h monitoring period for each housing condition. The humidity and temperature values were calculated at 2-h intervals and expressed as the percentage of relative humidity and °C, respectively.

### Horses’ behaviour

Individual horse behaviour was scored on a 1–10 scale to determine the horses’ stress level according to a previously published method in which 1–2 represents no stress and 8–10 indicates high stress (Young *et al.* 2012). The behaviours indicating stress included standing at the front of the stable, looking around, head descended below withers height, ears pricked or flattened, weaving behaviour, repeated tail swishing, scratching against stable walls or pawing at the ground with front legs. All nine horses were observed for behavioural changes in response to different housing conditions using internet protocol (IP) cameras (MC2MP-4CW, Goke Microelectronics, Hunan, China) stationed in the individual stables at a height of 2.5 m. The cameras were connected to a SAMSUNG Galaxy A70 (SM-A705F/DS, Samsung, Seoul, South Korea) and recorded consecutively for 12 h per housing condition. Horses’ behaviour was scored at 4-h intervals and evaluated by five equine veterinarians.

### Heart-rate measurement

Heart rates of six horses were monitored in the morning and afternoon, requiring approximately 4 h per measurement, using a heart-rate monitoring device (Seaver, Paris, France), as reported previously (Joongpan *et al.* 2021). Briefly, the girth sleeve containing the first electrode was fastened onto the horse’s girth using a custom-designed belt. The second electrode was then placed on the left wither and connected to the girth sleeve. Finally, the girth sleeve was paired with the Seaver application via a Bluetooth wireless interconnection and exported as an average heart rate for each horse. The average heart rate of individuals was evaluated in order to estimate the horses’ mean heart rate within the given periods in the two housing types.

### Blood cortisol concentration

Blood samples were collected from all horses at 0600, 1000, 1400 and 1800h while they were housed in the vector-protected barn without airflow manipulation, and at 0600, 0800, 1000, 1200, 1400, 1600 and 1800h while they were housed in the barn with airflow manipulation. Blood samples (5 mL) were taken from the jugular vein using 18-G needles connected to 5-mL syringes. The blood was allowed to clot in blood tubes containing a clot activator to obtain the serum. The sera were sent to a laboratory (Thai Vet Lab, Bangkok, Thailand) for analysis of cortisol concentration using a competitive chemiluminescent enzyme immunoassay (IMMULITE Analyzers, Siemens Healthineers, Erlangen, Germany). Blood cortisol levels were compared only at similar time-points between the two housing conditions.

### Statistical analysis

The statistical analyses were performed using GraphPad Prism (version 9.4.0; GraphPad Software, San Diego, CA, USA). The changes in stable humidity and temperature, blood cortisol level, heart rate and horses’ behavioural score in both housing conditions were evaluated using two-way analysis of variance (ANOVA) based on the general linear model (GLM), followed by Tukey’s *post hoc* test for comparisons within groups and the main row effect of the parameters. Šídák’s *post hoc* tests were alternatively implemented for comparisons between groups of each parameter. A paired *t*-test was applied to assess stable wind speed. Spearman’s rank-order correlation test was implemented to evaluate the correlation between stable humidity and temperature in both housing conditions. Data are expressed as mean (± SEM) and statistical significance was assigned at *P* < 0.05.

## Results

### Stable humidity and temperature

#### Stable humidity and temperature without ventilation adjustment

**Figure 3.**
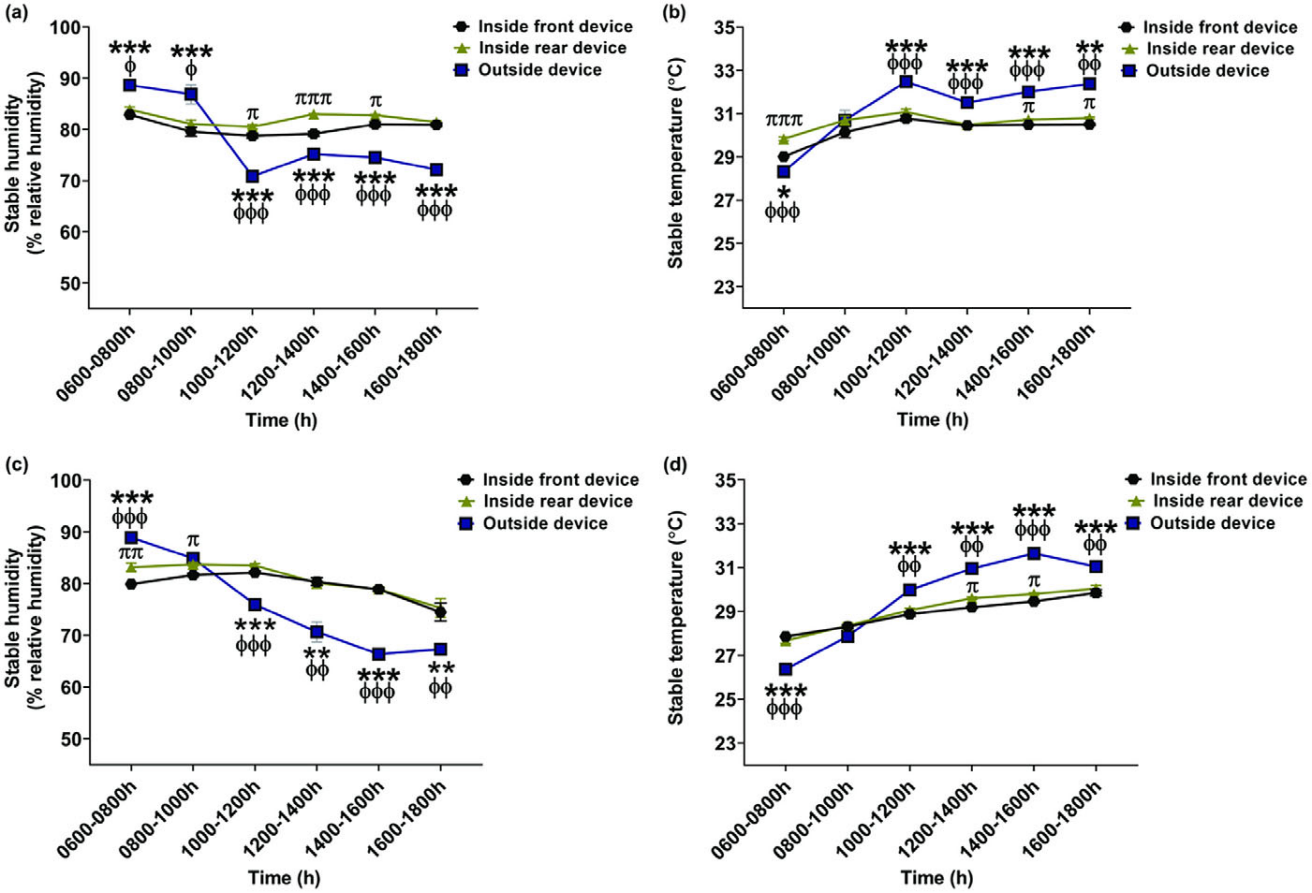
Line graphs representing the stable humidity and temperature inside and outside the barn during housing in the barn without (a) and (b) and with (c) and (d) ventilation adjustment. The humidity and temperature were recorded in 15-min intervals and evaluated during every 2-h period. The data were expressed as % relative humidity and °C, respectively. ^π^ *P* < 0.05, ^ππ^ *P* < 0.01 ^πππ^, *P* < 0.001 significant difference between the inside front and rear device. *P < 0.05, **P < 0.01, ***P < 0.001, significant difference between the inside front and outside device. ^ϕ^*P* < 0.05, ^ϕϕ^*P* < 0.01, ^ϕϕϕ^*P* < 0.001, significant difference between the inside rear and outside device.

Stable humidity and temperature without ventilation adjustment are shown in Table 1. Significant effects of device-installed position, time and device-installed position-by-time were observed (*P* < 0.0001 in all). Air velocity inside the barn was 0.32 (± 0.06) m s^–1^ (Table 2). In all periods, the humidity and temperature values were compared to the values at 0600–0800h (control). The inside humidity reached a peak at 0600–0800h and then markedly declined at 1000–1600h in the front (Fid) of the barn (1000–1400h; *P* < 0.01 and 1400–1600h; *P* < 0.05). The Fid humidity was not different at 1600–1800h. A decrease in the humidity in the rear (Rid) of the barn was only observed at 1000–1200h and 1600–1800h (*P* < 0.01 for both) (Table 1). Inside humidity measurements varied between the two devices throughout the experiment, and there was a large difference in stable humidity at 1000–1600h (1000–1200h and 1400–1600h; *P* < 0.05, 1200–1400h; *P* < 0.001) (Figure 3[a]). Although outside humidity (Od) was higher than inside humidity at 0600–1000h (Fid vs Od; *P* < 0.001, Rid vs Od; *P* < 0.05), it declined to its lowest value at 1000–1200h relative to inside humidity (*P* < 0.001 in both Fid and Rid vs Od). Subsequently, it increased but was still significantly lower than inside humidity at 1200–1800h (*P* < 0.001 for all pairs of comparisons) (Figure 3[a]).

**Table 1.** Stable humidity and temperature without ventilation adjustment.

Period (h)	Humidity and temperature measuring devices					
	Humidity (% relative value)			Temperature (°C)		
	Fid	Rid	Od	Fid	Rid	Od
0600–0800h	82.9 (± 0.8)	83.9 (± 0.5)	88.7 (± 0.7)	29.0 (± 1.1)	29.8 (± 0.1)	28.2 (± 0.2)
0800–1000h	79.6 (± 0.9)	81.1 (± 0.8)	86.9 (± 1.9)	30.1 (± 0.3)*	30.7 (± 0.2)*	30.7 (± 0.4)***
1000–1200h	78.8 (± 0.4)**	80.5 (± 0.4)**	70.8 (± 1.2)***	30.8 (± 0.1)***	31.1 (± 0.1)***	32.5 (± 0.2)***
1200–1400h	79.2 (± 0.4)**	83.0 (± 0.1)	75.2 (± 0.5)***	30.5 (± 0.1)***	30.5 (± 0.0)***	31.5 (± 0.1)***
1400–1600h	81.0 (± 0.5)*	82.8 (± 0.2)	74.5 (± 0.3)***	30.5 (± 0.0)***	30.7 (± 0.0)***	32.0 (± 0.0)***
1600–1800h	80.9 (± 0.4)	81.5 (± 0.2)**	72.2 (± 1.2)***	30.5 (± 0.1)***	30.8 (± 0.1)***	32.4 (± 0.2)***

The data were measured for 12 h and then evaluated at 2-h intervals.

Fid = Front inside humidity and temperature measuring device, Rid = Rear inside humidity and temperature measuring device, Od = Outside humidity and temperature measuring device.

* *P* < 0.05; significant difference from the value at 0600–0800h;

** *P* < 0.01; significant difference from the value at 0600–0800h;

*** *P* < 0.001; significant difference from the value at 0600–0800h.

**Table 2.** Wind speed inside the net-covered barn with and without ventilation adjustment.

Position		Wind speed inside the barn (m s^–1^)	
		(–) ventilation adjustment	(+) ventilation adjustment
Inside front stable		0.2	2.1
Barn corridor	front	0.5	2.3
	middle	0.4	2.5
	rear	0.2	2.5
Inside rear stable		0.3	3.2
Mean (± SEM)		0.32 (± 0.06)	2.40 (± 0.02)***

The data were recorded for 10 min at each position.

*** *P* < 0.001, significant difference of the values from (–) ventilation adjustment.

In contrast to the stable humidity, the stable temperature recorded in both Fid and Rid markedly increased at 0800–1800h (0800–1000h; *P* < 0.05 and 1000–1800 h; *P* < 0.001). In addition, the outside temperature rose considerably at 0800–1800h (*P* < 0.001) (Table 1). Even though the outside temperature was lower than the inside temperature at 0600–0800h (Fid vs Od; *P* < 0.05, Rid vs Od; *P* < 0.001) (Figure 3[b]), it increased sharply at 0800–1200h and reached a plateau higher than the inside temperature at 1200–1800h (*P* < 0.001 for both Fid and Rid vs Od, except at 1600–1800h, which was *P* < 0.01 for both Fid and Rid vs Od). A difference in the inside stable temperature was also noted at 0600–0800h (*P* < 0.001) and 1400–1600h (*P* < 0.05) (Figure 3[b]).

#### Stable humidity and temperature with ventilation adjustment

Stable humidity and temperature in response to ventilation adjustment within the barn are listed in Table 3. Significant effects of device-installed position, time and device-installed position-by-time were still observed (*P* < 0.0001 for all) in the barn with ventilation adjustment. Air velocity inside the barn with airflow manipulation was significantly greater than in the barn without airflow manipulation (*P* < 0.001) (Table 2). The humidity and temperature data in the periods were compared with those at 0600–0800h (control). The maximum inside humidity measured by Rid was observed at 0600–1200h. It subsequently decreased at 1400–1600h (*P* < 0.01) and 1600–1800h (*P* < 0.05). There was no difference in the inside humidity recorded by Fid during the day (Table 3). A peak in outside humidity was detected at 0600–0800h. It then decreased progressively from 1000h to the lowest value at 1600–1800h (*P* < 0.001) (Table 3). The outside humidity was significantly higher than the inside humidity at 0600–0800h (*P* < 0.001; Fid and Rid vs Od) (Figure 3[c]). It declined progressively until it was lower than inside humidity at 1000–1800h (1000–1200h and 1400–1600h; Fid and Rid vs Od; *P* < 0.001, 1200–1400h and 1600–1800h, Fid and Rid vs Od; *P* < 0.01). A difference in the inside humidity between the front and rear of the barn was only detected at 0600–0800h (*P* < 0.01) and 0800–1000h (*P* < 0.05) (Figure 3[c]).

**Table 3.** Stable humidity and temperature with ventilation adjustment.

Period (h)	Humidity and temperature recording devices					
	Humidity (% relative value)			Temperature (°C)		
	Fid	Rid	Od	Fid	Rid	Od
0600–0800h	79.9 (± 0.5)	83.2 (± 0.8)	88.9 (± 0.8)	27.9 (± 0.1)	27.7 (± 0.1)	26.3 (± 0.2)
0800–1000h	81.7 (± 0.3)	83.7 (± 0.4)	84.9 (± 1.2)	28.3 (± 0.1)*	28.4 (± 0.1)*	27.9 (± 0.3)*
1000–1200h	82.2 (± 0.3)	83.5 (± 0.3)	76.0 (± 0.6)***	28.9 (± 0.1)***	29.1 (± 0.1)***	30.0 (± 0.2)***
1200–1400h	80.4 (± 0.7)	80.1 (± 1.0)	70.7 (± 1.9)***	29.2 (± 0.0)***	29.6 (± 0.1)***	31.0 (± 0.2)***
1400–1600h	78.9 (± 0.3)	78.9 (± 0.5)**	66.4 (± 1.0)***	29.5 (± 0.1)***	29.8 (± 0.1)***	31.6 (± 0.2)***
1600–1800h	74.5 (± 1.7)	75.3 (± 1.8)*	67.3 (± 1.1)***	29.8 (± 0.2)***	30.0 (± 0.1)***	31.0 (± 0.2)***

The data were measured for 12 h and then evaluated at 2-h intervals.

Fid = Front inside humidity and temperature measuring device, Rid = Rear inside humidity and temperature measuring device, Od = Outside humidity and temperature measuring device

* *P* < 0.05; significant difference from the value at 0600–0800h.

** *P* < 0.01; significant difference from the value at 0600–0800h.

*** *P* < 0.001; significant difference from the value at 0600–0800h.

Inside and outside stable temperature markedly increased at 0800–1800h (0800–1000h; *P* < 0.05, 1000–1800h; *P* < 0.001) (Table 3). The outside temperature was lower than the inside temperature at 0600–0800h (Fid and Rid vs Od; *P* < 0.001) (Figure 3[d]). The outside temperature was significantly higher than that inside the barn at 1000–1800h (1000–1400h and 1600–1800h; Fid vs Od; *P* < 0.001 and Rid vs Od; *P* < 0.01, except at 1400–1600h, which was *P* < 0.001 for both Fid and Rid vs Od). The difference in the inside temperature between the front and rear of the barn was detected at 1200–1600h (*P* < 0.05) (Figure 3[d]).

### Correlation between stable humidity and temperature

**Figure 4.**
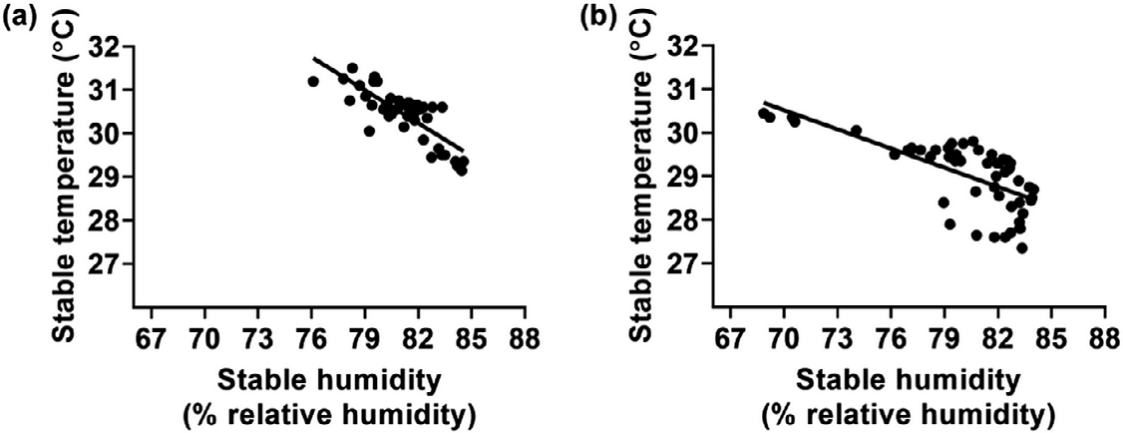
Negative correlation between stable humidity and temperature in (a) the vector-protected barn (*r* = –0.70; *P* < 0.0001) and (b) the vector-protected barn with ventilation adjustment (*r* = –0.74; *P* < 0.0001).

The correlation between stable humidity and temperature was determined according to a method described previously (Hinkle *et al.* 2003; Mukaka 2012). The stable humidity was highly negatively correlated with stable temperature in the vector-protected barn without airflow manipulation (*r* = –0.70) (Figure 4[a]) and the vector-protected barn with airflow manipulation (*r* = –0.74) (Figure 4[b]).

### Heart rate and horses’ behaviour

**Figure 5.**
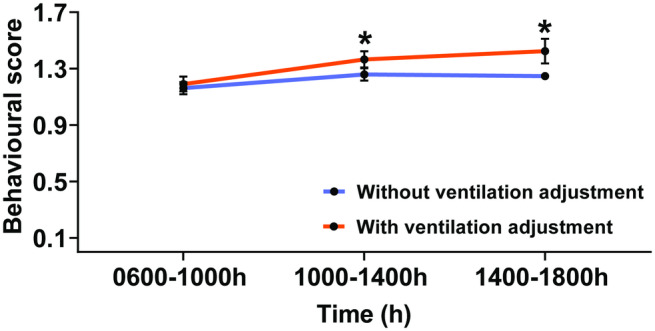
Mean (± SEM) of the behavioural score at 0600–1000h, 1000–1400h and 1400–1800h. *P < 0.05, significant difference from the value at 0600–1000h.

There was no significant effect of housing condition (*P* = 0.53), time (*P* = 0.13) or housing condition-by-time (*P* = 0.08) in the heart-rate measurement during housing in both conditions of the vector-protected barn. However, the effect of the individual horse was statistically significant (*P* < 0.01). Horses housed in the vector-protected barn without ventilation adjustment showed an increase in heart rate at 1400–1800h, compared to the value at 0700–1100h (*P* < 0.05), whereas no change in heart rate was shown in horses housed in the barn with ventilation adjustment (Table 4). Regarding the behavioural score, no significant effect of housing condition-by-time, housing condition, and the individual horse was detected (*P* = 0.06, *P* = 0.36 and *P* = 0.17, respectively). Only a main effect of time was observed during housing in the vector-protected barn (*P* < 0.05). The behavioural score increased at 1000–1400h (*P* < 0.05) and 1400–1800h (*P* < 0.05), compared with the value at 0600–1000h (Figure 5).

**Table 4.** Horses’ heart rate during housing in the net-covered barn with and without ventilation adjustment.

Period	Heart rate (bpm)	
	(–) ventilation adjustment	(+) ventilation adjustment
0700–1100h	36.83 (± 1.22)	36.80 (± 1.93)
1400–1800h	39.17 (± 1.25)*	36.60 (± 1.50)

* *P* < 0.05, significant difference from the value at 0700–1100h.

### Blood cortisol determination

**Figure 6.**
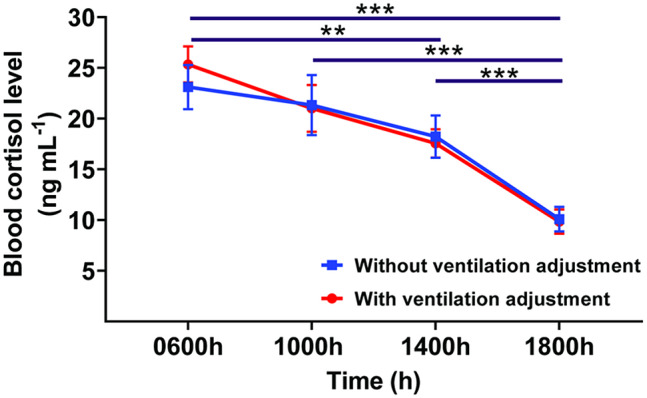
Mean (± SEM) serum cortisol levels at 0600, 1000, 1400 and 1800h. *P < 0.05, **P < 0.01, ***P < 0.001; significant difference between time-points.

There was no significant effect of housing condition (*P* = 0.89) or housing condition-by-time (*P* = 0.84); however, time (*P* < 0.0001) and individual horse (*P* < 0.05) effects were observed in the blood cortisol analyses. Blood cortisol level peaked at 0600h and markedly decreased at 1400h (*P* < 0.01), progressively reducing to the lowest value at 1800h (*P* < 0.001). Significant changes in cortisol level were also observed between 1000 and 1800h and between 1400 and 1800h (*P* < 0.001 in both) (Figure 6). Regarding the simple effect of time within each housing condition, the cortisol level still peaked at 0600h in both housing conditions. Horses housed in the barn with ventilation adjustment showed an earlier decline in cortisol level at 1400h (*P* < 0.05) that continued to decrease until 1800h (*P* < 0.001), whereas horses housed in the barn without ventilation adjustment showed a later decline in cortisol level at 1800h (*P* < 0.01) (Figure S2).

## Discussion

In the present study, stable humidity and temperature, heart rate, horses’ behaviour and cortisol release were determined in response to housing in a vector-protected barn with and without strategic ventilation adjustment. A main finding of the study was a wide range of internal humidity and temperature values at different positions in the vector-protected barn. Implementation of strategic ventilation adjustment in the barn led to less variability in inside stable humidity during the day than in the barn without strategic ventilation adjustment. Although there was no effect of housing condition or housing condition-by-time on changes in blood cortisol level in this study, we did observe an earlier decrease in cortisol release in horses housed in the barn with strategic ventilation adjustment compared to horses housed in the barn without the adjustment. An early reduction in cortisol release may indicate, at least in part, that the horses experienced minimal stress over time when housed in the ventilation-adjusted, vector-protected barn.

Since *Culicoides* midges’ activity peaks at sunset and sunrise (Mellor *et al.* 2000; Sanders *et al.* 2012), keeping the horse in the vector-protected housing during these times would be necessary to protect against AHS (Paton 1863). Stabling adjustment to prevent midges from entering the barn has been reported previously (Meiswinkel *et al.* 2000; Baker *et al.* 2015). Meiswinkel *et al.* (2000) demonstrated that accommodating horses at night in stables where the openings were covered with a fine-meshed green cloth could reduce the number of midges inside the stables (Meiswinkel *et al.* 2000). Furthermore, the application of commercial pyrethroid insecticides on the barn-installed net drastically decreased the possibility of viral transmission via midges (Baker *et al.* 2015). Therefore, accommodating horses in a vector-protected barn has enormous potential to reduce AHS viral infection and decrease the AHS fatality rate (Lincoln *et al.* 2015). Although horses in the outbreak area gained a substantial benefit from the net-covered barn’s vector-protection properties, they also suffered a considerable decrease in stable ventilation resulting from the closed barn system (Webster *et al.* 1987). Therefore, the small mesh net installed in the present study could decrease stable ventilation, possibly impairing horse welfare.

The construction of an additional vector-protected area connecting to the barn’s front gate and vector-protected arena facilitated the ventilation adjustment method and allowed the horses to perform light exercise. The air trapped in the stable could be released from the barn through the small mesh net of the established area while maintaining the vector-protection system. Although a highly negative correlation was observed in the present study between the changes in the inside humidity and temperature with and without airflow adjustment, there was no correlation between stable humidity and temperature from the inside to the outside of the net-covered barn. Notably, the inside-barn humidity was more homogeneous in response to strategic ventilation adjustments than when airflow was not adjusted. These data are supported by the lack of a difference in internal humidity values recorded by the two inside devices in the barn with ventilation adjustment compared to a marked difference in indoor humidity at 1000–1600h in the barn without ventilation adjustment (Figure 3[a], [c]).

Many dust and airborne microorganisms are generally found in horse barns (Woods *et al.* 1993; McGorum *et al.* 1998). A number of those air pollutants would be markedly increased in a barn with inadequate air ventilation (Clarke *et al.* 1987; McGorum *et al.* 1998). In addition, accumulation of ammonia, the waste product of urine and faeces, is substantially higher in an inadequately ventilated barn, regardless of bedding material (Curtis *et al.* 1996). Insufficient regular ventilation could also permit excessive moisture accumulation, leading to fungal growth inside the barn (Wathes 1994). Since air ventilation helps clear airborne particles and harmful gases from the barn (Curtis *et al.* 1996), a rapid increase in stable ventilation may diminish the accumulation of airborne ammonia within the barn (Wathes 1994). Although measurements of ammonia and other noxious gas concentrations were not available in this study, the increase in internal ventilation was intended to reduce indoor air pollution and potentially reduce horses’ discomfort during housing in the vector-protected barn.

Housing sytem-related stress responses in horses can be evaluated by their behaviour (Heleski *et al.* 2002; Søndergaard & Ladewig 2004; Clémence *et al.* 2020) and heart rate (Rivera *et al.* 2002; Harewood & McGowan 2005). Horses housed in individual boxes are likely to suffer social isolation and natural locomotive deprivation, thereby weakening the expression of natural behaviour (Rivera *et al.* 2002; Ruet *et al.* 2019). Moreover, the presence of windows, which allow interaction with the external environment during separate housing, has no apparent effect on horses’ welfare, even though management factors such as including straw bedding and reducing concentrated feed are somewhat beneficial (Ruet *et al.* 2019). The horse may still exhibit stereotypical stress-induced behaviour, such as crib-biting (Wickens & Heleski 2010) and weaving (Ninomiya *et al.* 2007), due to the lack of movement and social interaction with conspecifics (Sarrafchi & Blokhuis 2013). Despite an increase in the horses’ behavioural scores as the main effect of time, the horses in the present study did not show abnormal behaviour in either housing condition, as the behavioural scores indicated no stress (1–2), according to the method of Young *et al.* (2012). It is possible that social interaction among the horses in the barn still occurred, as they were all housed in sequential stables on both sides of the barn corridor, thereby facilitating their view of other horses. Furthermore, the limited observation period (12 h) may not have been adequate to observe the expression of stereotypical and abnormal behaviour. As the exhibition of stereotypes in a horse may not broadly correspond to its well-being, another behavioural indicator may be required for welfare determination (Mason & Latham 2004). Heart rate has sometimes been adopted, with other measures, to assess stress response in horses to a housing system (Rivera *et al.* 2002; Harewood & McGowan 2005). A heart rate increase would accompany stress-induced changes in sympathetic activity (Von Borell *et al.* 2007), which accounts for the fight-or-flight response (Jansen *et al.* 1995). Although, in the present study, a change in heart rate was detected in horses housed in the vector-protected barn without ventilation adjustment, it was not sufficient for determining the horses’ stress level. Another indicator should be considered to provide a clear determination of a horse’s welfare in response to housing in a vector-protected barn (Ruet *et al.* 2019).

Acute stress triggers the release of cortisol, an endocrine hormone produced via the hypothalamic-pituitary-adrenocortical axis that acts on the cell-mediated immune system (Padgett & Glaser 2003; Dhabhar 2009). Cortisol measurements have generally been used to evaluate stress conditions and horses’ welfare (Alexander *et al.* 1988; Cordero *et al.* 2012). Cortisol release could correlate with the stress experienced by the horse during road transportation (Schmidt *et al.* 2010b, c, d), exercise training (Linden *et al.* 1991; Marc *et al.* 2000; Schmidt *et al.* 2010a) and confinement (Alexander *et al.* 1988; Harewood & McGowan 2005). Theoretically, cortisol expression follows a diurnal rhythm throughout the day that is dependent on the adrenal cortex’s rhythmic stimulation (Giannetto *et al.* 2015). A daily change in cortisol release indicates early morning acrophase, and it gradually decreases until it reaches the lowest value in the evening (Weibel & Brandenberger 2002; Aurich *et al.* 2015; Giannetto *et al.* 2015). A disturbance in the horse’s cortisol circadian rhythm would indicate chronic stress and poor welfare (Möstl & Palme 2002). Notably, the cortisol level in this study demonstrated a diurnal rhythm similar to a previous report (Giannetto *et al.* 2015). We previously reported that the cortisol level peaked at 0600h and declined to its lowest level at 1800h in the net-covered barn (Joongpan *et al.* 2021). We suggested that short-term accommodation in the net-covered barn may not radically affect the horses’ circadian rhythm. Although no effect of housing condition or the housing condition-by-time on changes in cortisol level was observed in horses in the present study, an earlier significant decline in the cortisol level at 1400h that continued to decrease until 1800h was a noticeably positive impact on horses housed in the vector-protected barn with ventilation adjustment. Likewise, horses housed in the barn with ventilation adjustment showed the earliest decline in cortisol level at 1200h when the parameter was measured at 2-h intervals (Figure S3). As there was no other external stimulus that could have affected the horses’ stress response, an earlier decline in cortisol release may be due, at least in part, to an increase in air ventilation inside the barn.

The first study limitation was the fact that the horses were not allowed to paddock or live outside the facilities during the severe AHS outbreak in this region due to the threat of possible disease transmission. Consequently, the experiments were conducted while the horses were housed long term in vector-protected facilities throughout the study, which may have led to an increase in the stress levels of the horses studied. This factor may explain the lack of a housing effect on the decrease in blood cortisol level, even though an earlier decline in the cortisol level was found in horses in the barn with ventilation adjustment. Second, the front gate could not be opened earlier than 0800h and had to be closed before 1700h. This limitation was due to the overriding concern of viral transmission during midge feeding times. Despite the positive outcome of strategic ventilation adjustment in the vector-protected barn, challenging questions remain. Future research could determine whether adjusting stable ventilation prior to 0600h would result in a greater decline in cortisol level, or whether an increase in air velocity utilising additional mechanical ventilation could significantly reduce cortisol release. Although housing in a vector-protected barn can decrease viral transmission and horse fatality rate in the AHS outbreak area, further investigation is required to determine the effect on horses’ stress level of long-term living in the barn, even with airflow adjustment.

### Animal welfare implications

The main objective of this study was to evaluate whether the proposed stable management method improved horses’ welfare while retaining vector-protected facility characteristics. Although the housing condition did not change the overall stress level, as indicated by the cortisol concentration, an earlier decline in cortisol release, with no difference in heart rate, may indicate a positive impact of ventilation adjustment on the horses’ stress level. With limited options to reduce stress or discomfort in horses, these results could inform horse owners and the equestrian community on practical stable management to preserve, at least in part, the horse’s welfare while being housed in an unfamiliar manner in the AHS outbreak area.

## Supplementary material

For supplementary material accompanying this paper visit https://doi.org/10.1017/awf.2023.10. click here to view supplementary material
