# Supplementary material for: The impact of strategic ventilation adjustments on stress responses in horses housed full-time in a vector-protected barn during the African horse sickness outbreak in Thailand
Source: Anim Welf. 2023 Mar 23;32:e19. doi: 10.1017/awf.2023.10 (PMC10936309; doi:10.1017/awf.2023.10)
Supplement: Supplementary file 1 [file awfsup.zip › S0962728623000106sup001.pdf]

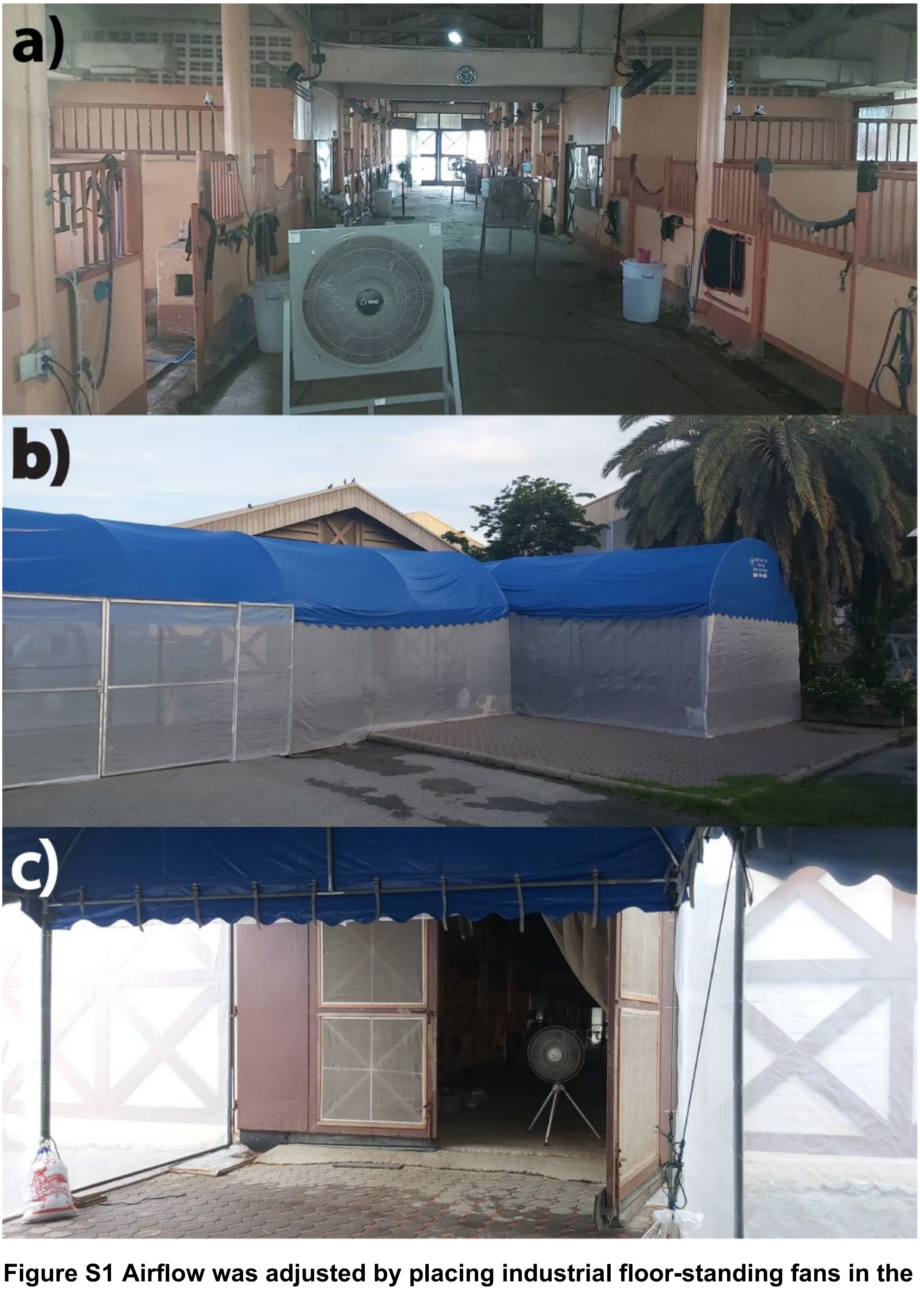

Figure S1 Airflow was adjusted by placing industrial floor-standing fans in the (a) the barn corridor. A temporary vector-protected area was established (b) and connected to the front gate to form (c) a closed protection system.
