# Supplementary material for: The impact of strategic ventilation adjustments on stress responses in horses housed full-time in a vector-protected barn during the African horse sickness outbreak in Thailand
Source: Anim Welf. 2023 Mar 23;32:e19. doi: 10.1017/awf.2023.10 (PMC10936309; doi:10.1017/awf.2023.10)
Supplement: Supplementary file 1 [file awfsup.zip › S0962728623000106sup002.pdf]

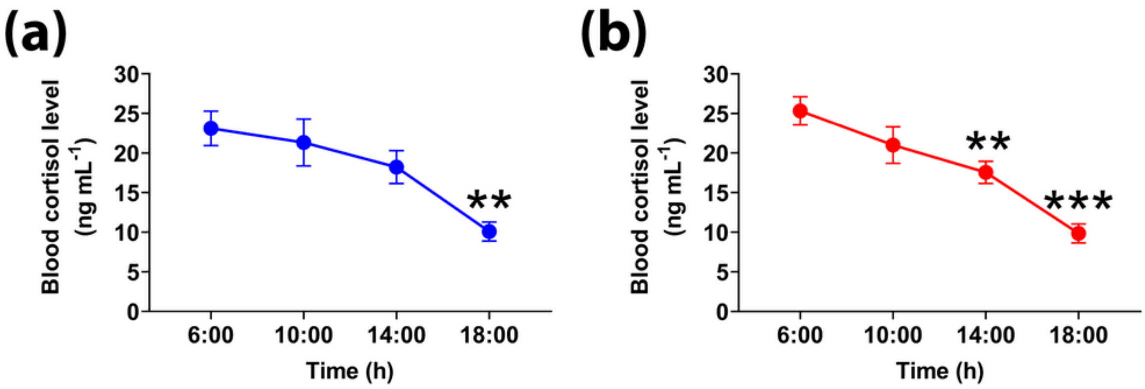

Figure S2 A comparison of blood cortisol variation during housing in the vector-protected barn (a) without and (b) with ventilation adjustment. Blood cortisol values were measured at 6:00 h, 10:00 h, 14:00 h, and 18:00 h. The cortisol levels at given time-points were compared to the value at 6:00 h (control).

$^{**}P < 0.01$, and $^{***}P < 0.001$, significant difference from the values at 6.00 h.
