# Supplementary material for: The impact of strategic ventilation adjustments on stress responses in horses housed full-time in a vector-protected barn during the African horse sickness outbreak in Thailand
Source: Anim Welf. 2023 Mar 23;32:e19. doi: 10.1017/awf.2023.10 (PMC10936309; doi:10.1017/awf.2023.10)
Supplement: Supplementary file 1 [file awfsup.zip › S0962728623000106sup003.pdf]

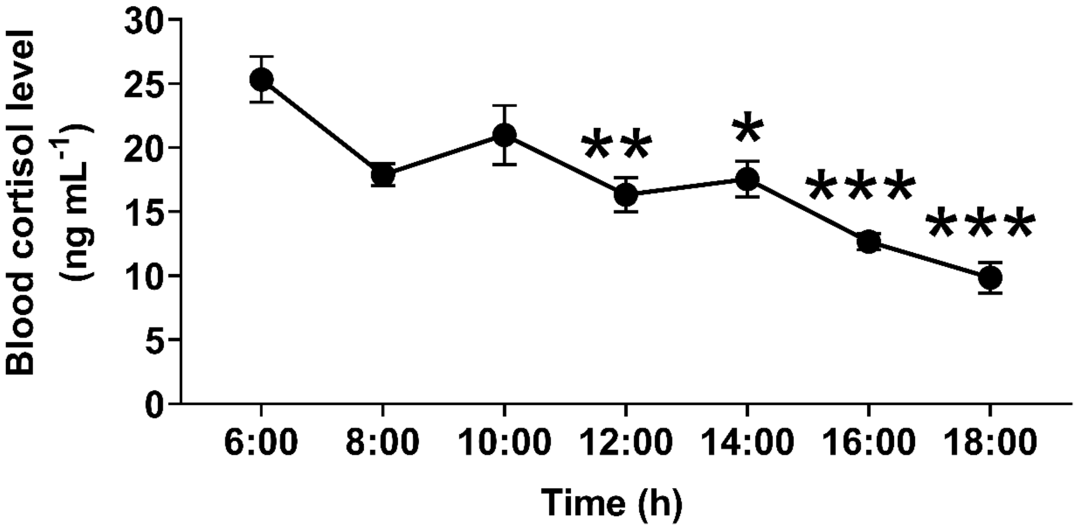

Figure S3 Variation in blood cortisol level in horses housed in the vector-protected barn with ventilation adjustment. Blood cortisol levels were measured at 6:00 h, 8:00 h, 10:00 h, 12:00 h, 14:00 h, 16:00 h and 18:00 h. The cortisol levels at given time points were compared to the value at 6:00 h (control). Blood cortisol level decreased significantly from 12:00 h to 18:00 h in horses housed in the vector-protected barn with strategic ventilation adjustment.
